# How marijuana use status affects responses to anti-marijuana messages

**DOI:** 10.26828/cannabis/2021.02.001

**Published:** 2021-05-19

**Authors:** Elise M. Stevens, Glenn Leshner, Amy M. Cohn, Seunghyun Kim, Theodore L. Wagener

**Affiliations:** 1 Department of Population and Quantitative Health Sciences, Division of Preventative and Behavioral Medicine, University of Massachusetts Medical School, Worcester, MA; 2 Gaylord College of Journalism and Mass Communication, The University of Oklahoma, Norman, OK; 3 Health Promotion Research Center, Stephenson Cancer Center, University of Oklahoma Health Sciences Center, Oklahoma City, OK; 4 Department of Pediatrics, College of Medicine, University of Oklahoma Health Sciences Center, Oklahoma City, OK; 5 Department of Marketing and Advertising, College of Business, University of Arkansas at Little Rock, Little Rock, AR; 6 Medical Oncology, The Ohio State University, Columbus, OH

**Keywords:** cannabis, use status, public health campaign, health communication, health messaging, perceptions of harm, message liking

## Abstract

**Background:**

The current study examined how cannabis use status impacts cognitive and emotional reactions to public health campaigns about cannabis, and the degree to which these reactions influence message likeability and attitudes about cannabis-related harms.

**Methods:**

In a between-subjects design, 252 subjects recruited via Amazon Mechanical Turk viewed six real-world cannabis education messages: three message themes (cognitive ability, driving, and health harms) from each of two real-world public campaigns. Subjects answered questions measuring their cognitive and emotional reactions to each message as well as message likeability and harm perceptions of cannabis. Analyses examined the mediating effects of message responsiveness on the association between baseline cannabis use (user vs non-user) with indices of liking and harm.

**Results:**

For all three message themes, informativeness ratings mediated the effect of cannabis user status on the outcomes of perceived harmfulness and message likeability. Specifically, cannabis users perceived cannabis as less harmful and reported all messages as less likeable compared to non-users, partly because they perceived the messages to be less informative than non-users. Surprisingly, users found some of the messages to be more pleasant, which was associated with increased perceptions of harm and message liking compared to non-users.

**Conclusions:**

Cannabis education campaigns that take into account differences in emotional and cognitive reactions by use experience, rather than use a “one size fits all” approach, could possibly maximally impact likeability and harm perceptions of these messages.

Given the rapid proliferation of new laws and policies surrounding cannabis use across the United States (Hartig & Geiger, 2018), the importance of conveying the risks of cannabis use to the public also increases (Monte et al., 2015). As of April 2021, 17 states have legalized recreational cannabis use, 36 states have legalized medical use, and 32 states have decriminalized use. In 2018 among Americans over the age of 12 years, 27.7 million reported using cannabis in the past 30 days (National Survey on Drug Use and Health, 2018). Because changes in cannabis policies could lead to increased acceptability of cannabis use and potentially, rises in the prevalence of initiation and use, it is important for public education efforts to be implemented in step with legalization in order to minimize potential public health harms from problematic cannabis use and related negative health outcomes (Monte et al., 2015).

Health risks associated with cannabis use include cancers (Cooper & Haney, 2009; Y.-H. J. Huang et al., 2015; Mehra et al., 2006), drug and alcohol use (Kam et al., 2009; White et al., 2005, 2006), lowered cognitive ability (Arria et al., 2013; D. Y. C. Huang et al., 2011) and mood disorders (Crane et al., 2015; Gage et al., 2015), as well as injury or death from car accidents (Blows et al., 2005). One effective way to raise awareness of these negative outcomes is through public health messaging campaigns, which reach many people at a relatively low cost (Farrelly et al., 2007; Goldman & Glantz, 1998). However, the empirically-tested public health campaigns that highlight the risks of cannabis use are nearly 20 years old (Palmgreen et al., 2001), which leaves an important gap in our understanding as to how best to convey these risks.

When designing messages within public health campaigns, there are various elements to consider. Specifically, it is important to understand the target audience’s cognitive and emotional responses to the message or messages within a campaign (Batra & Ray, 1986; Burke & Edell, 1986) including their liking and receptivity of the message (MacKenzie et al., 1986). These responses indicate how the audience evaluates the messages and how such messages could impact their future health behavior (Alvaro et al., 2013). McGuire’s Communication Persuasion Matrix (2001) shows that the steps in persuasion are as follows: step 1) exposure to the message, step 2) attention to the message, step 3) cognitive and emotional responses to the message, step 4) attitude towards the message and perceptions of the product, and step 5) product use or non-use; where step 4 is captured by likeability of the message (e.g., attitudes towards the message) and harm perceptions of cannabis (e.g., perceptions of the product). This study employs steps one through four to understand how cannabis education messages impact individuals’ responses to these messages. This study will inform which messages are best suited for interventions and thus be used in subsequent studies about product use (step 5). Specifically, this study examined how cognitive and emotional responses to a message (e.g., measured by pleasantness, unpleasantness, arousal, informativeness; step 3) mediate the association between cannabis use status and attitude toward the message and perceptions of the product (e.g., message liking, perceived harmfulness of cannabis; step 4).

Previous research has illustrated how cognitive and emotional responses outlined by step 3 in the persuasion matrix can impact perceptions and liking (step 4 in the persuasion matrix), which in turn impact use or non-use of the product (step 5). For example, one meta-analysis examining health messages found that messages that elicited an unpleasant emotional response (step 3 in the persuasion matrix), triggered perceptions of the product (step 4), and eventually had the ability to impact behavior change, step 5 (Witte & Allen, 2000). In addition, a study that examined perceptions of harm about Lyme Disease after viewing a health message about the Lyme Disease vaccine showed that increased harm perceptions of the disease (step 4) impacted engagement in getting the vaccine (e.g., step 5 - participating in the health behavior; Brewer et al., 2004). With regard to message liking, advertising studies for everyday products such as toothpaste have shown that liking of the message/advertisement was an important indicator of future positive behavior change/buying the product (step 4 and 5; MacKenzie et al., 1986). In summary, understanding how audiences might respond to health messages about cannabis is imperative to spreading awareness about the risk of use.

Based on health communication literature, two key factors may help to determine optimal message content in cannabis public health messages: 1) theme of the message (e.g., addiction, cessation, health or social consequences; Beaudoin, 2002) and 2) whether or not the intended audience has used or has had experience with the health behavior in question – in this case, having ever used or currently using cannabis (Cho et al., 2016; Dillard & Shen, 2005; National Cancer Institute, 2008; Wakefield et al., 2013). First, understanding how audiences react to different message themes can help campaign designers select optimal message content. Emotions, both positive and negative, can effectively change attitudes and behavior, and different types of message themes can elicit those emotions (Dillard & Nabi, 2006). Thus, it is important that messages elicit strong emotions, which can trigger beliefs (e.g., greater liking, increased perceptions of harm; Brewer et al., 2004; MacKenzie et al., 1986), and not the emotions that will likely deter people from the processing and remembering the message (e.g., disliking the message; Brewer et al., 2004; MacKenzie et al., 1986). While no study has examined the themes of public health messages focused on cannabis, one review of anti-tobacco messaging studies found that different themes (e.g., addiction, industry manipulation, cessation, health consequences of tobacco) were effectively employed to cater to the target audience (Beaudoin, 2002). Results of this review showed that messages targeted at adults were mostly fear appeals and focused on long-term health consequences (Beaudoin, 2002).

The second factor that may play a role in audience reactions is prior or current experience with the behavior highlighted in the message, in this case, cannabis use status. For example, tobacco studies have shown that cigarette smokers are less inclined to react positively to anti-smoking messages and can feel annoyed or defensive (Wolburg, 2006), while non-smokers have been shown to react positively (National Cancer Institute, 2008). It is possible that individuals who have experience using cannabis, either in the past or who currently use, may react differently to messages about cannabis-related health risks than individuals who have never used cannabis. Thus, the first step in implementing cannabis public health messages is to understand responses to messages with regard to theme and use status. If use status is an important factor in message responsiveness in terms of perceived harmfulness of cannabis and message liking, this would mean that anti-cannabis messages should not be developed as a “one size fits all” approach, but rather, messages should be created to separately target users and non-users, perhaps with themes and message types that are unique to their experiences with the product.

This study proposes to examine cognitive and emotional reactions to public health education messages about cannabis so that existing and future campaigns can have a greater impact. Specifically, the current study examined whether cognitive and emotional reactions mediate differences in cannabis users’ and non-users’ responses to real-world cannabis public health campaigns, spanning three message themes: 1) cognitive ability, (2) driving fatalities, and 3) health harms. After viewing each message, participants were asked to evaluate their emotional state, arousal, perception of the message (how informative the message was), liking of the message, and harm perceptions of cannabis use. Understanding audience reactions to currently existing messages may help identify areas of strength and weakness for effective message development that target these risk perceptions. Based on previous research, we hypothesized that non-users would show evidence of more positive responses to messages (e.g., increased message liking and increased perceptions of harm) and that increased perceptions of informativeness and unpleasantness would in turn be associated with increased message liking and increased perceptions of harm.

## METHODS

Participants (*N* = 258) were cannabis users and non-users recruited from Amazon Mechanical Turk (MTurk) during the summer of 2017. MTurk samples have been found to be similar to nationally representative survey samples (Coppock, 2019; Kees et al., 2017; Walters et al., 2018). Inclusion criteria were: aged 18 or older and located within the United States (as verified by IP address). The survey link was posted on MTurk and users could opt-in to answer a survey advertised as, “We are researchers studying the perceptions of health messages. We are inviting you to participate in our online survey. Participation in this research includes viewing health messages and then answering questions about your perceptions of those messages and attitudes after viewing the messages. It will take you approximately 25 minutes.” After consent was obtained, participants answered questions about demographics (age, sex, and race) and current cannabis use (“somedays,” “every day,” “not at all”). Then, participants viewed six anti-cannabis print messages obtained from real world campaigns in the United States. After viewing each message, participants answered questions in the following domains: pleasant and unpleasant affect, arousal, perceived message, perceived message informativeness, message liking, and perceived harmfulness of cannabis. All participants saw all messages, and messages were presented to participants in a random order. Participants spent on average, 8.04 minutes (*SD* = 4.98 minutes) completing the survey. They could view the messages for as long as they wanted. Procedures were approved by the University of Oklahoma’s institutional review board. Participants were paid $0.50 for completing the task, which is slightly higher than a typical survey of this length on MTurk (Buhrmester et al., 2015).

### Anti-Cannabis Messages

Messages were obtained from two real world public health campaigns: the “Do the Math” print campaign developed by the Liberty Alliance for Youth, a coalition in Liberty, MO (http://libertyalliance4youth.com/), and the “Spread the Facts” print campaign developed for adolescents by the National Institute on Drug Abuse for Teens (https://teens.drugabuse.gov/). Participants viewed three messages from each of the two campaigns, and both campaigns provided a message from each of the following themes: cognitive ability, driving ability, and health harms associated with cannabis use. Cognitive ability messages focused on drops in IQ from cannabis use. Driving messages focused on car accidents due to being under the influence of cannabis. Health harms messages focused on medically necessary admissions for drug treatments and increases in anxiety and depression due to cannabis use. Messages from the “Do the Math” campaign contained stick figure drawings and messages from the “Spread the Facts” campaign contained real life imagery. See Table 1 for a description of the stimuli.

This study aimed to combat single-message design effects by having participants in the study view two different messages per message theme (one message per theme from each campaign), an approach that aligns with other published studies (e.g., Goodall et al., 2013; Jensen, 2008; Kim et al., 2012; Lee et al., 2011). This allowed us to attribute differences due to the theme category and not attributes in a single message. It also allowed us to strengthen our ability to generalize the results to the real world (Leshner, 2013; O’Keefe, 2003; Reeves & Geiger, 1994; Tao & Bucy, 2007; Thorson et al., 2012).

### Measures

#### Independent Variable

##### Cannabis use status.

Participants answered the question “How frequently do you use marijuana?” Response options were “somedays,” “every day,” and “not at all.” Participants who answered “somedays” or “every day” were categorized as cannabis users and those who answered “not at all” were categorized as non-users. For mediation models (described below) users were coded as 1 and non-users were coded as 0.

#### Covariates

##### Demographics.

Age, sex, and race were used as covariates. Race was assessed with the following categories: Asian, Black/African American, Native American/Alaskan, Pacific Islander, White/Caucasian, More than one/Other. Race was recoded into the three largest categories. Asian, White, and Other.

#### Mediators

##### Pleasant affect.

Pleasant affect was measured post-message viewing, using the question, “How pleasant did this message make you feel?” on a scale from 1 (not at all) to 7 (extremely). This measure was adapted from an existing measure to assess positive affect (Bradley & Lang, 1994; Clayton et al., 2018; Watson et al., 1988) and was averaged for each message theme.

##### Unpleasant affect.

Unpleasant affect was measured using the question, “How unpleasant did this message make you feel?” on a scale from 1 (not at all) to 7 (extremely). This measure was adapted to assess negative affect (Bradley & Lang, 1994; Clayton et al., 2018; Watson et al., 1988) was averaged for each message theme.

##### Arousal.

Arousal was measured using the question, “How did this message make you feel?” on a scale from 1 (calm) to 7 (excited). This was an adapted measure (Bradley & Lang, 1994; Clayton et al., 2018; Watson et al., 1988) with higher scores reflecting greater arousal. It is important to note that arousal is typically measured separately from pleasant and unpleasant affect as a separate construct (Watson & Tellegen, 1985). Items were averaged for each message theme.

##### Message informativeness.

Message informativeness was measured with the question, “Please rate the message on the following: This message was informational” on a scale from 1 (not at all) to 7 (extremely). Items were averaged for each message theme.

#### Dependent Variables

##### Message Liking.

Participants were asked how much they liked the message they just saw, “Please rate this message on a scale from 1 (disliked it very much) to 7 (liked it very much),” which was adapted from previous work (Unger et al., 1995). Items were averaged for each message theme.

##### Perceived Harmfulness of Cannabis.

Perceived harmfulness of cannabis was assessed using the question, “How harmful do you think marijuana is to your health?” on a scale from 1 (not harmful at all) to 5 (extremely harmful) (National Institutes of Health [NIH] and the U.S. Food and Drug Administration [FDA], 2013). Items were averaged for each message theme.

#### Data Analysis

**Figure 1.**
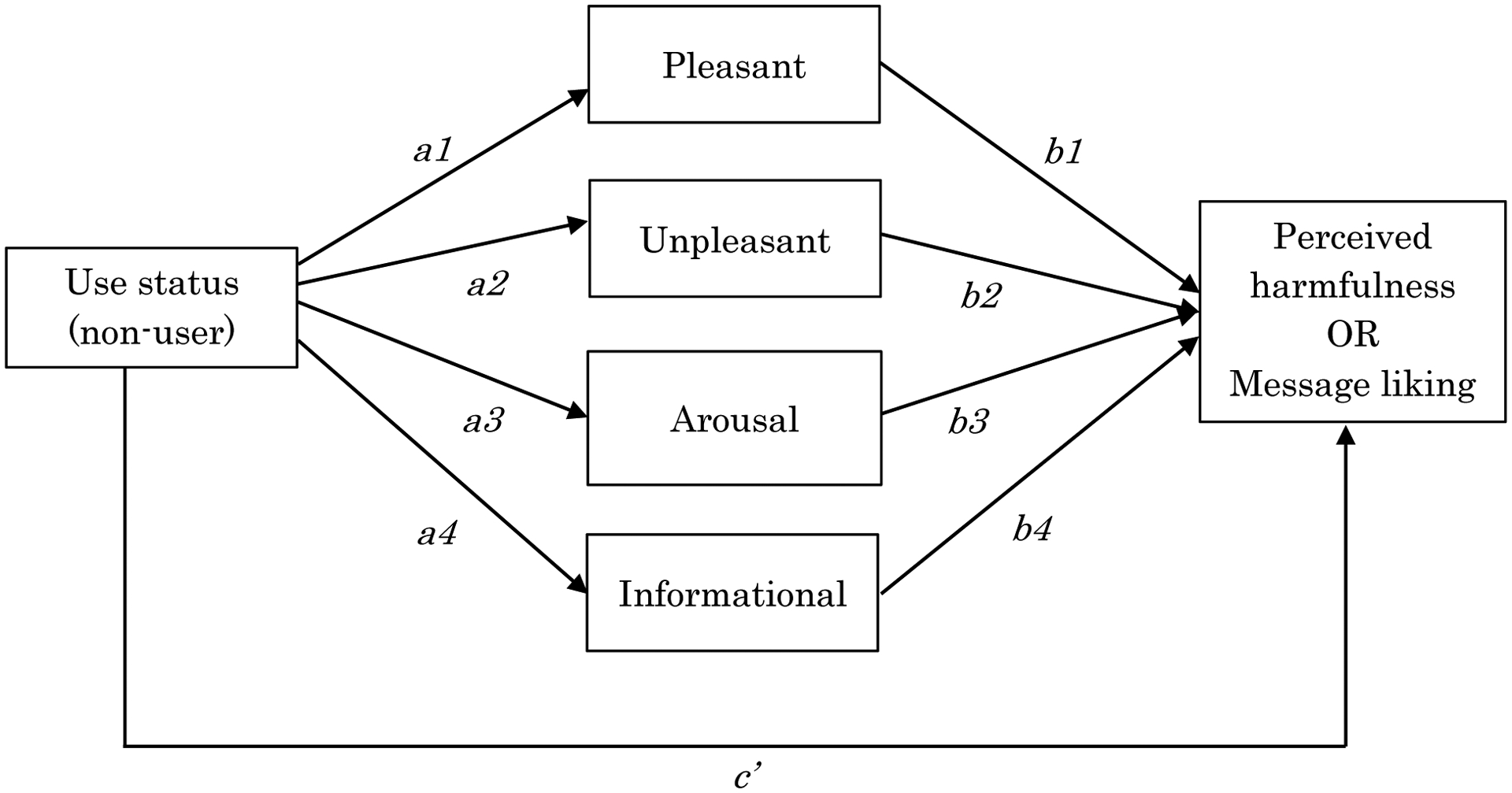
Conceptual multiple mediation model Note: c’ represents the direct effect of use status on a dependent variable.

Figure 1 shows the conceptual mediation model of the association between cannabis use status and perceived harmfulness of cannabis and message liking as the primary dependent variables of interest, with pleasant, unpleasant, arousal, and message informativeness, as mediators. All models controlled for age, sex, and race. Analyses were conducted in IBM’s SPSS 24 using Andrew Hayes’ Process v3.3 Model Number 4. Direct effects of use status on the two dependent variables (perceived harmfulness and message liking) for each of the three message themes (cognitive ability, driving, and health harms) are discussed first. It is important to note that direct effects of use status on each of the outcome variables control for the effects of the four mediators. Then, the indirect effects of the four mediating variables (pleasant, unpleasant, arousal, informativeness) on each dependent variable are discussed. Separate mediation models were conducted for each message theme. In PROCESS, the A*B paths were used to estimate the indirect effects of cannabis use status on either perceived harmfulness or message liking across the four potential mediating variables (pleasant, unpleasant, arousal, informativeness). P-values of less than 0.05 were considered statistically significant and 95% confidence intervals are reported for each model for the indirect effects.

## RESULTS

### Participants

Participants (*N* = 252) ranged in age from 18 to 76 (*M* = 37.24, *SD* = 11.57) and were 48.8% female (*n* = 126; nine individuals did not report their sex). The majority of participants were White (67.8%), followed by Asian (16.7%). Fifty-eight percent (58.5%) (*n* = 151) were cannabis non-users, while 39.1% were “somedays” or “everyday” users (*n* = 101; six individuals did not report their use status).

Users and non-users differed on demographics, where users were younger [*F*(1, 249) = 46.56, *p* < .001], more likely to be male (χ^2^ = 8.22, *p* < .01), and more likely to be White (χ^2^ = 7.11, *p* < .05). See Table 2 for a summary of means and standard deviations by use status for each message theme.

### Direct Effects of Cannabis Use Status on Perceived Harmfulness and Message Liking

Table 3 shows the results of the mediation models. For each of the three message themes, users reported significantly lower perceived harm of cannabis, post-message viewing, than did non-users (all *p*’s < .05; Table 3). In addition, there were significant negative direct effects for cognitive ability and health harms themes on message liking.

Specifically, for perceived harmfulness, cannabis users rated cannabis as significantly less harmful than non-users in every message theme category: cognitive ability messages (b = −0.47, *p* < .01), the driving messages (b = −0.36, *p* < .05), and the health harms messages (b = −0.53, *p* < .001). Sex was significantly associated with perceived harmfulness (b = −29, *p* < .05), such that men rated cannabis more harmful than women in cognitive ability message category.

Similar associations were found for the direct effect of cannabis use status on message liking for two of the three message themes. Specifically, cannabis users reported significantly lower message liking for cognitive ability messages (b = −0.49, *p* < .05) and for health harms messages (b = −0.53, *p* < .001). Sex (b = −.27, *p* < .05) was significantly associated with message liking such that men liked the cognitive ability messages more. Age was also significantly associated with liking of the message, such that older participants liked the messages more in two categories (cognitive ability: b = .01, *p* < .01; driving: (b = .01, *p* < .001).

### Mediation Models

Six multiple mediation models were tested. The A paths (i.e., the path from use status to each mediating variable) for each message theme are shown in Table 4. The B paths (i.e., the paths from each mediator variable to each of the two dependent variables) for each message theme are shown in Table 3. Also shown in Table 3 are the indirect effects of cannabis use status on each dependent variable through the mediators (A*B paths).

### Indirect Effects of Cannabis Use Status on Perceived Harmfulness of Cannabis

#### Cognitive ability messages.

There was an indirect effect of ratings of message informativeness on the association between cannabis use status and perceived harmfulness (b = −0.27, 95%CI = [−0.44, −0.13]). Users rated the cognitive ability messages as less informative than non-users (b = −1.09, *p* < .001), and ratings of informativeness were positively associated with perceived harmfulness (b= 0.25, *p* < .001). Therefore, compared to non-users, users reported significantly lower perceived harm after viewing the cognitive ability messages, partly because they rated the messages as less informative.

#### Driving ability messages.

Similar to the cognitive ability messages, there was an indirect effect of ratings of informativeness on the association between cannabis use status and perceived harmfulness (b = −0.35, 95%CI = [−0.54, −0.19]). Users rated the driving messages as less informative than non-users (b = −1.25, *p* < .001), and ratings of message informativeness were positively associated with perceived harmfulness (b = 0.28, *p* < .001). Therefore, users (compared to non-users) perceived cannabis to be less harmful after viewing the driving messages, partly because they rated the messages as less informative.

There was also an indirect effect of pleasantness ratings on the association between cannabis use status and perceived harmfulness (b = 0.12, 95%CI = [0.01, 0.18]). Users (compared to non-users) reported more pleasantness in response to the driving messages (b = 0.70, *p* < .01) and pleasantness was positively associated with perceived harmfulness (b = 0.12, *p* < .01). Therefore, users perceived cannabis as more harmful than non-users, partly because they felt more pleasant after viewing the messages.

#### Health harms messages.

Similar to both the cognitive ability and the driving messages, there was an indirect effect of ratings of message informativeness on the association between use status and perceived harmfulness for the health harms messages (b = −0.30, 95%CI = [−0.48, −0.15]). Users rated the health harms messages as less informative than non-users (b = −0.98, *p* < .001), but ratings of informativeness were positively associated with perceived harmfulness (b = 0.31, *p* < .001). Therefore, users (compared to non-users) perceived cannabis as less harmful, partly because they rated the message as less informative.

### Message Liking as the Outcome

#### Cognitive ability messages.

Ratings of informativeness (b = −0.52, 95%CI = [−0.78, −0.28]) and pleasantness (b = 0.24, 95%CI = [0.07, 0.44]) both emerged as significant mediators of the association between cannabis use status and message liking. Specifically, users rated the cognitive ability messages as less informative than non-users (b = −1.09, *p* < .001), and message informativeness was positively associated with message liking (b = 0.48, *p* < .001). Therefore, users liked the cognitive ability messages less than non-users, partly because they rated the messages as less informative.

The indirect effect of pleasantness showed a different pattern. Users (compared to non-users) had greater feelings of pleasantness when viewing the cognitive ability messages (b = 0.72, *p* < .01), and pleasantness was positively associated with message liking (b = 0.33, *p* < .001). Therefore, users liked the cognitive ability messages more than non-users, partly because they felt more pleasant.

#### Driving ability messages.

For the driving messages, ratings of informativeness (b = −0.66, 95%CI = [−0.99, −0.39]) and pleasantness (b = 0.26, 95%CI = [0.07, 0.46]) both emerged as significant mediators of the association between cannabis use status and message liking. Specifically, users rated the driving messages as less informative than non-users (b = −1.25, *p* < .001), and perceived informativeness was positively associated with message liking (b = 0.53, *p* < .001). Therefore, users liked the driving messages less than non-users, partly because they rated the messages as less informative.

The indirect effect of pleasantness showed a different pattern. Users (compared to non-users) reported more pleasantness in the driving message category (b = 0.70, *p* < .01) and pleasantness was positively associated with message liking (b = 0.37, *p* < .001). Therefore, users liked the driving messages more than non-users partly because they reported feeling more pleasant.

#### Health harms messages.

Similar to both the cognitive ability and the driving messages, informative ratings emerged as a mediator of the association between use status and message liking (b = −0.30, 95%CI = [−0.75, −0.24]). Users rated the health harms messages as less informative than non-users (b = −0.98, *p* < .001), but informative ratings were positively associated with message liking (b = 0.31, *p* < .001). Therefore, users perceived the health harms messages as more likeable than non-users, partly because they rated the message as less informative. See Table 3.

## DISCUSSION

The current study is one of the first to examine how cannabis use status impacts important cognitive and emotional reactions to public health campaigns about cannabis, and the degree to which these reactions influence message likeability and attitudes about cannabis-related harms. Consistent with previous work (Kilmer et al., 2007), across all three message themes (cognitive ability, driving, and health harms), cannabis users reported lower ratings of perceived harm than non-users. Because we did not measure and control for individual differences in cannabis harm perceptions prior to message viewing, we cannot determine if this effect was a result of message viewing or pre-existing differences in harm perceptions. Similar direct effects of cannabis use status were found for message liking, wherein users, overall, reported lower message likeability than non-users for the cognitive ability and health harms messages. These direct effects remained despite significant indirect effects, suggesting that there may be other mediators, not measured in this study, that could better explain these associations. These may include other factors such as comorbid alcohol or tobacco use, depression, and anxiety as well as degree of knowledge about cannabis. However, these results do support the notion that use status is an important factor to consider when designing health messages about cannabis.

A primary goal of this study was to examine cognitive and affective mechanisms that underlie responsivity and effectiveness of health messages about cannabis. We approached this by considering several possible mediating variables that may influence the relationship between cannabis use status and two important message outcomes—perceived cannabis harmfulness and message liking. Several noteworthy indirect effects were found. Across all three message themes, users reported all messages as being less informative than non-users, and lower perceived informativeness was associated with lesser perceived harm of cannabis use and message liking. One possible explanation for these mediating results could be that users think they already know the negative impacts cannabis use has on cognitive ability, driving, and on one’s health, and information in these messages was merely re-iterating existing knowledge, or did not provide new or novel facts. Another possible explanation could be that cannabis users may not believe the risks noted in the messages because these risks may be at odds with their personal experiences with cannabis use (e.g., I have driven after smoking cannabis and have never been in a car accident). Although both explanations are speculative, it is noteworthy that users’ lack of perceived informativeness of message content was consistently associated with both reduced perceived cannabis harmfulness and reduced message liking. To enhance the effectiveness of such messages, it would be important that message content is novel. Interestingly, we found that for the driving messages, reported pleasantness showed a positive indirect effect, such that users thought the messages were more pleasant than non-users, which then was associated with increased perceived harmfulness and increased message liking. This same result was also found in the cognitive ability messages associated with message liking. One explanation for this may be that feeling pleasant has the capability to “broaden and build” one’s mind (Fredrickson, 2001), thus resulting in more positive perceptions of the message and the information conveyed in the message. Taken together, presenting pleasantly emotionally charged education messages with novel information may be an effective approach to enhancing cannabis harm perceptions when targeted specifically toward cannabis users. Messages that do not invoke a high level of emotionality (Biener et al., 2000, 2006; Dillard & Peck, 2000; Pechmann & Reibling, 2006) and those that contain less “newsworthy” and perhaps outdated facts on cannabis use may not be interesting or mentally stimulating enough to capture the attention or retain in memory as long (Peters et al., 2019), and thus have little impact on attitudes about the risks of using.

Although this study is the first to examine cannabis public health campaigns, it does align with past work focused on health campaigns for other health behaviors, such as flossing, alcohol use, and tobacco use (Witte & Allen, 2000; Wolburg, 2006). In the current study, we found that non-users liked the messages more than users, which is in line with some tobacco research comparing smokers and non-smokers (National Cancer Institute, 2008; Wolburg, 2006). More work needs to be done to determine which message themes increase message receptiveness for users and change actual behavior.

Not only did this study highlight the importance of theme and use, it also identified possible mediating variables, particularly how informative and how emotional viewers perceive the message to be. However, this study is not without its limitations. First, this study utilized MTurk making it a convenience sample that may not be generalizable to the American population (Kraemer et al., 2017). For instance, the percentage of cannabis users in this study was higher than the national average (National Survey on Drug Use and Health, 2018). Although this study was focused on examining various responses to cannabis public health messages, actual behavior or behavioral intentions following message viewing were not assessed. In addition, ethnicity was not assessed either. Future studies should examine how cognitive and affective responses to cannabis health messages may impact behavioral measures as well as more demographics of the audience. Although considered a strength, this study examined real-world campaigns that had already been developed. As a result, we were unable to experimentally manipulate message content to determine which specific aspects of content could optimally impact harm perceptions and message likeability. Future studies should isolate specific features of each message to better understand which features impact responses to the messages. Lastly, while we tested mediation, our models align with statistical mediation, not causal mediation, because the mediators and the outcomes were assessed at the same time. Overall, this study highlighted the importance of theme and cannabis use status when designing and testing the potential impact of cannabis public health messages on harm perceptions and message liking. Findings highlight that different message themes resonate to a different degree with adults. Further, different messages appear to have unique effects on cognitive and emotional processing. Taken together, our findings suggest that cannabis education campaigns that incorporate several different message themes, rather than use a “one size fits all” approach, could maximally impact likeability of these messages and harm perceptions associated with cannabis.

This study is the first study to our knowledge to examine responses to cannabis public health messages to understand the impact of theme and cannabis use status and explore several mediating mechanisms. The relationships analyzed in this paper are important mechanisms along the pathway from message exposure to behavior change. Future studies should continue to examine this area as legalization of cannabis spreads across the U.S.

## Figures and Tables

**Figure 2.**
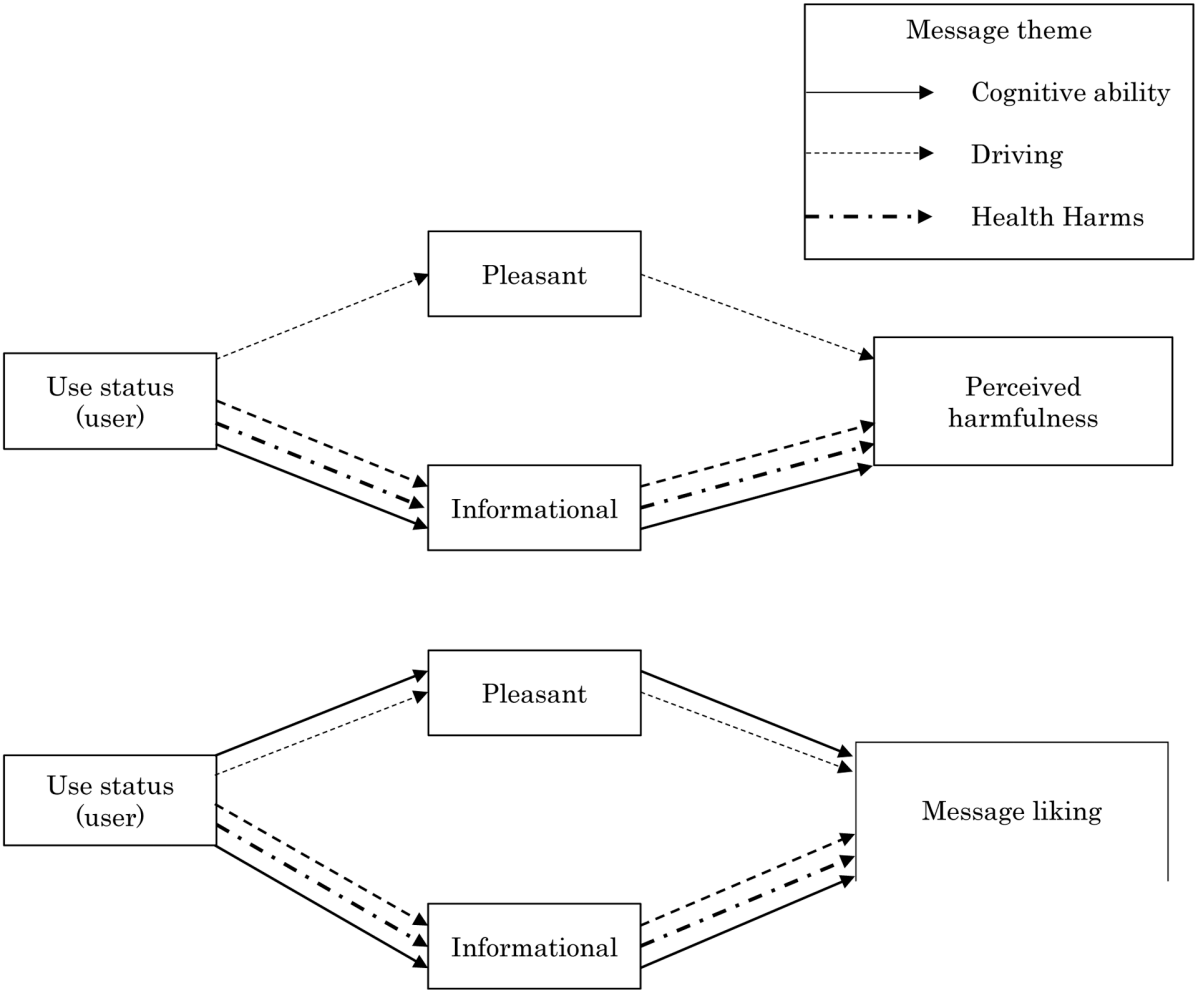
Indirect effects of use status on perceived harmfulness and message liking Note: Only statistically significant indirect paths are shown (*p* < .05).

**Table 1.** Cannabis messages

Cognitive Ability	Cognitive Ability
**Text**: Marijuana. It lowers your intelligence. New research shows a permanent drop in IQ when marijuana is used regularly before the age of 18.	**Text**: Do the Math. Fact: Marijuana has been shown to permanently drop IQ by an average of 8 points.
**Image**: Photograph of woman holding her hand to her face.	**Image**: A math equation showing a drawing of a cannabis leaf and a plus sign with a drawing of a human head and brain on the other side. After the equals sign, there is an image of the letters “IQ” that points downard.
Driving	Driving
**Text**: Marijuana. It impairs driving. In the United States, marijuana is the most commonly identified illegal drug in fatal crashes (14% of drivers).	**Text**: Do the Math. Fact: Marijuana is the most common illegal drug found in drivers who are in car accidents.
**Image**: Photograph of car crash scene with an ambulance and emergency helicopter.	**Image**: A math equation showing a drawing of a cannabis leaf and a plus sign with a drawing of a human head and brain on the other side. After the equals sign, there is an drawing of a car driving into a lamppost.
Health Harms	Health Harms
**Text**: Marijuana. It’s hamrful to youth. About 68% of all medically necessary drug treatment admissions for youth ages 12–17 are for marijuana.	**Text**: Do the Math. Fact: Marijuana has been shown to double the risk of anxiety and depression.
**Image**: Photograph of a young man sitting in an empty hallway with his elbows resting on his knees and his head hanging.	**Image**: A math equation showing a drawing of a cannabis leaf and a plus sign with a drawing of a human head and brain on the other side. After the equals sign, there is stick figure human with a thought bubble that reads, “anxiety & depression.”

**Table 2.** Means for measures by message theme and cannabis use status

	Message Themes								
	Cognitive			Driving			Health Harms		
Measures	Non-user	User	η_p_^2^	Non-user	User	η_p_^2^	Non-user	User	η_p_^2^
Pleasant	2.24 (1.32)	3.10 (1.87)	.07***	2.10 (1.37)	3.04 (1.79)	.08***	2.28 (1.39)	2.99 (1.90)	.04**
Unpleasant	4.54 (1.67)	4.05 (1.84)	.02*	4.90 (1.60)	4.15 (1.67)	.05**	4.58(1.70)	4.16 (1.81)	.01
Arousal	3.60 (1.52)	3.72 (1.71)	.00	4.08 (1.58)	3.92 (1.77)	.00	3.59 (1.51)	3.80 (1.55)	.00
Informational	4.94 (1.56)	3.90 (1.86)	.08***	5.09 (1.42)	3.93 (1.82)	.11***	4.93 (1.44)	3.99 (1.88)	.07***
Liking	4.40(1.51)	3.65 (1.79)	.05**	4.39 (1.50)	3.77 (1.81)	.03**	4.31 (1.60)	3.58 (1.79)	.04**
Harmful	3.07 (1.18)	2.42 (1.13)	.07***	3.03 (1.17)	2.38 (1.11)	.07***	3.09 (1.19)	2.40 (1.14)	.09***

Note: Cell entries are means; standard deviations are in parentheses. Pleasant, unpleasant, and informational ranged on a scale from 1 “not at all” to 7 “extremely.” Arousal ranged from 1 “calm” to 7 “excited.” Message liking ranged from 1 “disliked it very much” to 7 “liked it very much.” Perceived harmfulness ranged from 1 “no harm at all” to 5 “extremely harmful.”

* *p* < .05,

** *p* < .01,

*** *p* < .001.

**Table 3.** Mediators and indirect effects predicting perceived harmfulness and message liking

	Outcome											
	Perceived Harmfulness						Message Liking					
Message Theme	Cognitive Ability		Driving		Health Harms		Cognitive Ability		Driving		Health Harms	
PATH B	b	SE	b	SE	b	SE	b	SE	b	SE	b	SE
b1 (pleasant)	.10*	.05	.12**	.05	.03	.04	.33***	.05	.37***	.05	.03	.04
b2 (unpleasant)	.07	.05	.06	.05	.04	.04	−.03	.05	.05	.06	.04	.04
b3 (arousal)	.08	.06	.07	.05	.17**	.05	.19**	.06	−.001	.06	.17**	.05
b4 (informational)	.25***	.04	.28***	.05	.31***	.04	.48***	.05	.53***	.06	.31***	.04
A*B	b	SE	b	SE	b	SE	b	SE	b	SE	b	SE
pleasant	.07	.05	.08*	.04	.02	.03	.24*	.09	.26*	.10	.02	.03
unpleasant	−.02	.03	−.04	.04	−.02	.02	.01	.03	−.04	.05	−.01	.02
arousal	.01	.03	−.005	.02	.04	.04	.04	.5	.000	.02	.04	.04
informational	−.27*	.08	−.35*	.09	−.30*	.08	−.52*	.13	−.66*	.15	−.30*	.09
Direct Effects	b	SE	b	SE	b	SE	b	SE	b	SE	b	SE
Cannabis use status	−.47**	.15	−.36*	.15	−.53***	.14	−.49**	.16	−.19	.18	−.53***	.14

*Note*. All models controlled for age, sex, and race. Cannabis use status was coded “ 0” for non-users and “1” for users.

* p < .05

** p < .01

*** p < .001

**Table 4.** Use status predicting mediators

Message Theme	Cognitive Ability		Driving		Health Harms	
	b	SE	b	SE	b	SE
**PATH A**						
a1 (pleasant)	.72**	.23	.70**	.22	.61**	.23
a2 (unpleasant)	−.37	.25	−.72**	.24	−.35	.25
a3 (arousal)	.23	.23	−.01	.24	.25	.22
A4 (informational)	−1.09***	.24	−1.25***	.23	−.98***	.23

*Note*. All models controlled for age, sex, and race.

* p < .05

** p < .01

*** p < .001

## References

[R1] Alvaro EM, Crano WD, Siegel JT, Hohman Z, Johnson I, & Nakawaki B (2013). Adolescents’ attitudes toward antimarijuana ads, usage intentions, and actual marijuana usage. Psychology of Addictive Behaviors, 27(4), 1027–1035. 23528197 10.1037/a0031960 PMC4480868

[R2] Arria AM, Garnier-Dykstra LM, Cook ET, Caldeira KM, Vincent KB, Baron RA, & O’Grady KE (2013). Drug use patterns in young adulthood and post-college employment. Drug and Alcohol Dependence, 127(1–3), 23–30. 22743161 10.1016/j.drugalcdep.2012.06.001 PMC3463732

[R3] Batra R, & Ray ML (1986). Affective responses mediating acceptance of advertising. Journal of Consumer Research, 13(2), 234.

[R4] Beaudoin CE (2002). Exploring antismoking ads: Appeals, themes, and consequences. Journal of Health Communication, 7(2), 123–137. 12049421 10.1080/10810730290088003

[R5] Biener L, McCallum-Keeler G, & Nyman AL (2000). Adults’ response to Massachusetts anti-tobacco television advertisements: impact of viewer and advertisement characteristics. Tobacco Control, 9(4), 401–407. 11106710 10.1136/tc.9.4.401 PMC1748390

[R6] Biener L, Reimer RL, Wakefield M, Szczypka G, Rigotti NA, & Connolly G (2006). Impact of smoking cessation aids and mass media among recent quitters. American Journal of Preventive Medicine, 30(3), 217–224. 16476637 10.1016/j.amepre.2005.10.026

[R7] Blows S, Ivers RQ, Connor J, Ameratunga S, Woodward M, & Norton R (2005). Marijuana use and car crash injury. Addiction, 100(5), 605–611. 15847617 10.1111/j.1360-0443.2005.01100.x

[R8] Bradley MM, & Lang PJ (1994). Measuring emotion: The self-assessment manikin and the semantic differential. Journal of Behavior Therapy and Experimental Psychiatry, 25(1), 49–59. 7962581 10.1016/0005-7916(94)90063-9

[R9] Brewer NT, Weinstein ND, Cuite CL, & Herrington JE (2004). Risk perceptions and their relation to risk behavior. Annals of Behavioral Medicine, 27(2), 125–130. 15026296 10.1207/s15324796abm2702_7

[R10] Buhrmester M, Kwang T, & Gosling SD (2015). Amazon’s Mechanical Turk: A new source of inexpensive, yet high-quality data? In Methodological issues and strategies in clinical research (4th ed.). (pp. 133–139). American Psychological Association. 10.1177/1745691610393980 26162106

[R11] Burke MC, & Edell JA (1986). Ad reactions over time: Capturing changes in the real world. Journal of Consumer Research, 13(1), 114.

[R12] Cho YJ, Thrasher JF, Swayampakala K, Yong H-H, McKeever R, Hammond D, Anshari D, Cummings KM, & Borland R (2016). Does reactance against cigarette warning labels matter? Warning label responses and downstream smoking cessation amongst adult smokers in Australia, Canada, Mexico and the United States. PLOS ONE, 11(7), e0159245. 27411100 10.1371/journal.pone.0159245 PMC4943644

[R13] Clayton RB, Lang A, Leshner G, & Quick BL (2018). Who fights, who flees? An integration of the LC4MP and psychological reactance theory. Media Psychology, 1–27.

[R14] Cooper ZD, & Haney M (2009). Comparison of subjective, pharmacokinetic, and physiological effects of marijuana smoked as joints and blunts. Drug and Alcohol Dependence, 103(3), 107–113. 19443132 10.1016/j.drugalcdep.2009.01.023 PMC2776770

[R15] Coppock A (2019). Generalizing from survey experiments conducted on Mechanical Turk: A replication approach. In Political Science Research and Methods (Vol. 7, Issue 3, pp. 613–628). Cambridge University Press.

[R16] Crane NA, Langenecker SA, & Mermelstein RJ (2015). Gender differences in the associations among marijuana use, cigarette use, and symptoms of depression during adolescence and young adulthood. Addictive Behaviors, 49, 33–39. 26036667 10.1016/j.addbeh.2015.05.014 PMC4478142

[R17] Dillard JP, & Nabi RL (2006). The persuasive influence of emotion in cancer prevention and detection messages. Journal of Communication, 56(s1), S123–S139.

[R18] Dillard JP, & Peck E (2000). Affect and persuasion. Communication Research, 27(4), 461–495.

[R19] Dillard JP, & Shen L (2005). On the nature of reactance and its role in persuasive health communication. Communication Monographs, 72(2), 144–168.

[R20] Farrelly MC, Hussin A, & Bauer UE (2007). Effectiveness and cost effectiveness of television, radio and print advertisements in promoting the New York smokers’ quitline. Tobacco Control, 16 Suppl 1(Suppl 1), i21–3. 18048625 10.1136/tc.2007.019984 PMC2598515

[R21] Fredrickson BL (2001). The role of positive emotions in positive psychology: The broaden-and-build theory of positive emotions. American Psychologist, 56(3), 218–226. 11315248 10.1037//0003-066x.56.3.218 PMC3122271

[R22] Gage SH, Hickman M, Heron J, Munafò MR, Lewis G, Macleod J, & Zammit S (2015). Associations of cannabis and cigarette use with depression and anxiety at age 18: Findings from the Avon Longitudinal Study of Parents and Children. PLOS ONE, 10(4), e0122896. 25875443 10.1371/journal.pone.0122896 PMC4395304

[R23] Goldman LK, & Glantz SA (1998). Evaluation of antismoking advertising campaigns. JAMA, 279(10), 772. 9508154 10.1001/jama.279.10.772

[R24] Goodall CE, Slater MD, & Myers TA (2013). Fear and anger responses to local news coverage of alcohol-related crimes, accidents, and injuries: Explaining news effects on policy support using a representative sample of messages and people. Journal of Communication, 63(2), 373–392. 23729838 10.1111/jcom.12020 PMC3665547

[R25] Hartig H, & Geiger A (2018). About six-in-ten Americans support marijuana legalization. Pew Research http://www.pewresearch.org/fact-tank/2018/10/08/americans-support-marijuana-legalization/

[R26] Huang DYC, Evans E, Hara M, Weiss RE, & Hser Y-I (2011). Employment trajectories: Exploring gender differences and impacts of drug use. Journal of Vocational Behavior, 79(1), 277–289. 21765533 10.1016/j.jvb.2010.12.001 PMC3134335

[R27] Huang Y-HJ, Zhang ZF, Tashkin DP, Feng B, Straif K, & Hashibe M (2015). An epidemiologic review of marijuana and cancer: An update. Cancer Epidemiology Biomarkers & Prevention, 24(1), 15–31. 10.1158/1055-9965.EPI-14-1026 PMC4302404 25587109

[R28] Jensen JD (2008). Scientific uncertainty in news coverage of cancer research: Effects of hedging on scientists and journalists credibility. Human Communication Research, 34(3), 347–369.

[R29] Kam JA, Matsunaga M, Hecht ML, & Ndiaye K (2009). Extending the Theory of Planned Behavior to Predict Alcohol, Tobacco, and Marijuana Use Among Youth of Mexican Heritage. Prevention Science, 10(1), 41–53. 18985451 10.1007/s11121-008-0110-0

[R30] Kees J, Berry C, Burton S, & Sheehan K (2017). An Analysis of Data Quality: Professional Panels, Student Subject Pools, and Amazon’s Mechanical Turk. Journal of Advertising, 46(1), 141–155.

[R31] Kilmer JR, Hunt SB, Lee CM, & Neighbors C (2007). Marijuana use, risk perception, and consequences: Is perceived risk congruent with reality? Addictive Behaviors, 32(12), 3026–3033. 10.1016/j.addbeh.2007.07.009 17822856

[R32] Kim HS, Bigman CA, Leader AE, Lerman C, & Cappella JN (2012). Narrative health communication and behavior change: The influence of exemplars in the news on intention to quit smoking. Journal of Communication, 62(3), 473–492. 22736808 10.1111/j.1460-2466.2012.01644.x PMC3377164

[R33] Kraemer JD, Strasser AA, Lindblom EN, Niaura RS, & Mays D (2017). Crowdsourced data collection for public health: A comparison with nationally representative, population tobacco use data. Preventive Medicine, 102, 93–99. 28694063 10.1016/j.ypmed.2017.07.006 PMC5557015

[R34] Lee S, Cappella JN, Lerman C, & Strasser AA (2011). Smoking cues, argument strength, and perceived effectiveness of antismoking PSAs. Nicotine and Tobacco Research, 13(4), 282–290. 21330273 10.1093/ntr/ntq255 PMC3066405

[R35] Leshner G (2013). The Basics of Experimental Research in Media Studies. In The International Encyclopedia of Media Studies (pp. 236–254). John Wiley & Sons, Ltd.

[R36] MacKenzie SB, Lutz RJ, & Belch GE (1986). The role of attitude toward the ad as a mediator of advertising effectiveness: A test of competing explanations. Journal of Marketing Research, 23(2), 130.

[R37] Mehra R, Moore BA, Crothers K, Tetrault J, & Fiellin DA (2006). The association between marijuana smoking and lung cancer. Archives of Internal Medicine, 166(13), 1359. 16832000 10.1001/archinte.166.13.1359

[R38] Monte AA, Zane RD, & Heard KJ (2015). The implications of marijuana legalization in Colorado. JAMA, 313(3), 241. 25486283 10.1001/jama.2014.17057 PMC4404298

[R39] National Cancer Institute. (2008). The Role of the Media in Promoting and Reducing Tobacco Use NCI TOBACCO CONTROL MONOGRAPH SERIES. Smoking and Tobacco Control Monograph, 19(07–6242.). https://cancercontrol.cancer.gov/brp/tcrb/monographs/19/m19_complete.pdf

[R40] National Institutes of Health (NIH) and the U.S. Food and Drug Administration (FDA). (2013). Population Assessment of Tobacco and Health (PATH) Study, PATH Wave 1 Youth Extended Interview 7.5, 2013, Ad Exposure Section.

[R41] National Survey on Drug Use and Health. (2018). 2018 NSDUH Annual National Report | CBHSQ. Annual Report. https://www.samhsa.gov/data/report/slides-2018-nsduh-annual-national-report

[R42] O’Keefe DJ (2003). Message properties, mediating states, and manipulation checks: Claims, evidence, and data analysis in experimental persuasive message effects research. Communication Theory, 13(3), 251–274.

[R43] Palmgreen P, Donohew L, Lorch EP, Hoyle RH, & Stephenson MT (2001). Television campaigns and adolescent marijuana use: tests of sensation seeking targeting. American Journal of Public Health, 91(2), 292–296. http://www.ncbi.nlm.nih.gov/pubmed/11211642 11211642 10.2105/ajph.91.2.292 PMC1446528

[R44] Pechmann C, & Reibling ET (2006). Antismoking advertisements for youths: an independent evaluation of health, counter-industry, and industry approaches. American Journal of Public Health, 96(5), 906–913. 16571709 10.2105/AJPH.2004.057273 PMC1470598

[R45] Peters E, Shoots-Reinhard B, Evans AT, Shoben A, Klein E, Tompkins MK, Romer D, & Tusler M (2019). Pictorial warning labels and memory for cigarette health-risk information over time. Annals of Behavioral Medicine, 53(4), 358–371. 29947729 10.1093/abm/kay050 PMC6289901

[R46] Reeves B, & Geiger S (1994). Designing experiments that assess psychological responses. In Lang A (Ed.), Measuring psychological responses to media (pp. 165–180). LEA.

[R47] Tao C-C, & Bucy EP (2007). Conceptualizing media stimuli in experimental research: Psychological versus attribute-based definitions. Human Communication Research, 33(4), 397–426.

[R48] Thorson E, Wicks R, & Leshner G (2012). Experimental methodology in journalism and mass communication research. Journalism & Mass Communication Quarterly, 89(1), 112–124.

[R49] Unger JB, Johnson CA, & Rohrbach LA (1995). Recognition and liking of tobacco and alcohol advertisements among adolescents: Relationships with susceptibility to substance use. Preventive Medicine, 24(5), 461–466. 8524720 10.1006/pmed.1995.1074

[R50] Wakefield MA, Bowe SJ, Durkin SJ, Yong H-H, Spittal MJ, Simpson JA, & Borland R (2013). Does tobacco-control mass media campaign exposure prevent relapse among aecent quitters? Nicotine & Tobacco Research, 15(2), 385–392. 22949574 10.1093/ntr/nts134 PMC4288108

[R51] Walters K, Christakis DA, & Wright DR (2018). Are Mechanical Turk worker samples representative of health status and health behaviors in the U.S.? PLOS ONE, 13(6), e0198835. 29879207 10.1371/journal.pone.0198835 PMC5991724

[R52] Watson D, Clark LA, & Tellegen A (1988). Development and validation of brief measures of positive and negative affect: The PANAS Scales. Journal of Personality and Social Psychology, 54, 1063–1070. 3397865 10.1037//0022-3514.54.6.1063

[R53] Watson D, & Tellegen A (1985). Toward a consensual structure of mood. Psychological Bulletin, 98(2), 219–235. 3901060 10.1037//0033-2909.98.2.219

[R54] White HR, Labouvie EW, & Papadaratsakis V (2005). Changes in substance use during the transition to adulthood: A comparison of college students and their noncollege age peers. Journal of Drug Issues, 35(2), 281–306.

[R55] White HR, McMorris BJ, Catalano RF, Fleming CB, Haggerty KP, & Abbott RD (2006). Increases in alcohol and marijuana use during the transition out of high school into emerging adulthood: The effects of leaving home, going to college, and high school protective factors. Journal of Studies on Alcohol, 67(6), 810–822. 17060997 10.15288/jsa.2006.67.810 PMC2314672

[R56] Witte K, & Allen M (2000). A meta-analysis of fear appeals: Implications for effective public health campaigns. Health Education and Behavior, 27, 591–615. 11009129 10.1177/109019810002700506

[R57] Wolburg JM (2006). College students’ response to antismoking messages: Denial, defiance, and other boomerang effects. Journal of Consumer Affairs, 40, 294–323.

